# Non-myeloablative conditioning is sufficient to achieve complete donor myeloid chimerism following matched sibling donor bone marrow transplant for myeloproliferative leukemia virus oncogene (*MPL*) mutation-driven congenital amegakaryocytic thrombocytopenia: Case report

**DOI:** 10.3389/fped.2022.903872

**Published:** 2022-07-28

**Authors:** Joseph Hai Oved, Yash B. Shah, Kimberly Venella, Michele E. Paessler, Timothy S. Olson

**Affiliations:** ^1^Pediatric Transplantation and Cell Therapy, MSK Kids, New York, NY, United States; ^2^Cell Therapy and Transplant Section, Division of Pediatric Oncology, Children’s Hospital of Philadelphia, Philadelphia, PA, United States; ^3^Department of Pathology, Children’s Hospital of Philadelphia, Philadelphia, PA, United States

**Keywords:** congenital amegakaryocytic thrombocytopenia, bone marrow transplant, aplastic anemia, *MPL*, case report

## Abstract

**Background:**

Congenital amegakaryocytic thrombocytopenia (CAMT) is a rare platelet production disorder caused mainly by loss of function biallelic mutations in myeloproliferative leukemia virus oncogene (*MPL*), the gene encoding the thrombopoietin receptor (TPOR). Patients with *MPL*-mutant CAMT are not only at risk for life-threatening bleeding events, but many affected individuals will also ultimately develop bone marrow aplasia owing to the absence of thrombopoietin/TPOR signaling required for maintenance of hematopoietic stem cells. Curative allogeneic stem cell transplant for patients with CAMT has historically used myeloablative conditioning; however, given the inherent stem cell defect in *MPL*-mutant CAMT, a less intensive regimen may prove equally effective with reduced morbidity, particularly in patients with evolving aplasia.

**Methods:**

We report the case of a 2-year-old boy with *MPL*-mutant CAMT and bone marrow hypocellularity who underwent matched sibling donor bone marrow transplant (MSD-BMT) using a non-myeloablative regimen consisting of fludarabine, cyclophosphamide, and antithymocyte globulin (ATG).

**Results:**

The patient achieved rapid trilinear engraftment and resolution of thrombocytopenia. While initial myeloid donor chimerism was mixed (88% donor), due to the competitive advantage of donor hematopoietic cells, myeloid chimerism increased to 100% by 4 months post-transplant. Donor chimerism and blood counts remained stable through 1-year post-transplant.

**Conclusion:**

This experience suggests that non-myeloablative conditioning is a suitable approach for patients with *MPL*-mutant CAMT undergoing MSD-BMT and is associated with reduced risks of conditioning-related toxicity compared to traditional myeloablative regimens.

## Introduction

Congenital amegakaryocytic thrombocytopenia (CAMT) is a rare autosomal recessive inherited bone marrow failure syndrome (iBMFS) most commonly caused by pathogenic variants in *MPL*, the gene which encodes the thrombopoietin receptor (TPOR) (1). *MPL*-mutant CAMT can be caused by bi-allelic nonsense mutations in *MPL* that cause a complete absence of TPOR, or by missense mutations in the extracellular domain of TPOR, which can cause more indolent disease progression (2). Mutations in other genes comprising the TPO signaling axis can also cause CAMT (3, 4).

Patients with *MPL*-mutant CAMT generally present with bleeding symptoms within the first year of life. Patients may be managed initially with supportive measures, such as antifibrinolytic agents, to prevent routine mucocutaneous bleeding, but may require platelet transfusions to prevent catastrophic bleeds in patients at high risk for bleeding. While evolution to myelodysplasia and leukemia is uncommon in patients with *MPL*-mutant CAMT, most patients will progress to trilineage aplasia by 5 years of age (2). While gene therapy approaches are under development for *MPL*-mutant CAMT (5, 6), the only current clinically available curative therapy is allogeneic hematopoietic stem cell transplant (AlloSCT). AlloSCT for CAMT has traditionally been performed using fully myeloablative conditioning based on total body irradiation (TBI) or busulfan, or by using a melphalan-based reduced-intensity approach (7–9), even for patients who have evolving aplasia. These TBI-, busulfan-, or melphalan-based approaches expose patients to short-term mucosal, pulmonary, and hepatic toxicity, as well as long-term effects including infertility (10, 11). Recent evidence in other iBMFs, such as telomere biology disorders, has demonstrated that due to the growth disadvantage of recipient-derived hematopoietic stem cells (HSC), low-intensity, immune suppression-focused conditioning regimens can be used to produce durable donor engraftment with chimerism that improves over time (12). Because preclinical models of TPOR deficiency demonstrate a reduction in long-term hematopoietic stem cells (13) and because the natural history of aplasia development in *MPL*-mutant CAMT suggests that autologous reconstitution after alloSCT would be unlikely; myeloablative agents that target recipient HSC ablation may not be required in conditioning regimens for *MPL*-mutant CAMT. Here, we describe the successful use of a non-myeloablative conditioning regimen [as previously defined in a retrospective analysis of alloSCT for CAMT (14)], similar to that used for patients with acquired aplastic anemia (where ablation of host HSC is not required) (15, 16), in the setting of MSD-BMT for a 2-year-old patient with *MPL*-mutant CAMT who had evolving aplasia at the time of transplant. Success with our patient using this approach suggests that non-myeloablative conditioning could be used in other patients with *MPL*-mutant CAMT to spare toxicity while maintaining efficacy compared to historical myeloablative regimens. These data augment a previously reported retrospective analysis of alloSCT in patients with CAMT, in which 6/86 patients received a non-myeloablative conditioning regimen (14) and add additional granularity of the patient course, molecular studies, transplant characteristics, and chimerism data in a patient treated with a non-myeloablative conditioning regimen.

## Case presentation

A male with severe thrombocytopenia, petechiae, and bruising was the product of a 44-year-old G16P12 mother with a known antiphospholipid syndrome. He was tested for TORCH infections, anti-phospholipid antibodies, and neonatal alloimmune thrombocytopenia and was treated with IVIG and multiple platelet transfusions but with no improvement. An initial bone marrow aspirate performed in the first months of life was cellular, with markedly decreased megakaryocytes of variable morphology. Gene sequencing revealed pathogenic homozygous c.79+2T>A frameshift mutations in *MPL*, resulting in truncation near the n-terminal portion of TPOR (1) consistent with *MPL*-mutant CAMT. This mutation has been described as a founder mutation in the Ashkenazi Jewish community, with a carrier frequency of 1 in 75 and predicted incidence of 1 in 22,500 pregnancies (17). This mutation results in severely impaired Thrombopoietin/TPOR signaling on both megakaryocyte progenitors and HSC (18, 19), thereby, constraining megakaryopoiesis and HSC maintenance despite the high levels of serum thrombopoietin (2).

The patient required eight platelet transfusions in the first 2 months of life. Thereafter, his platelet count remained around 20 K/μL over the next year, and he required only 2 additional pre-transplant platelet transfusions due to episodes of hematochezia and prolonged epistaxis.

Upon referral to our institution at 1 year of age for curative therapy consultation, a CBC showed a platelet count of 17 k/μL, hemoglobin (Hgb) of 11.8 g/dL, and a normal absolute neutrophil count (ANC) of 4,130/μL. An 18-year-old male sibling was identified as a 10/10 HLA-matched donor. Since the patient was not requiring recurrent platelet transfusions at this time and repeat bone marrow aspirate with biopsy at 15 months of age showed preserved cellularity (60–70%) without dysplasia and a normal karyotype, we elected to continue close monitoring while the family considered curative therapy options.

**FIGURE 1:**
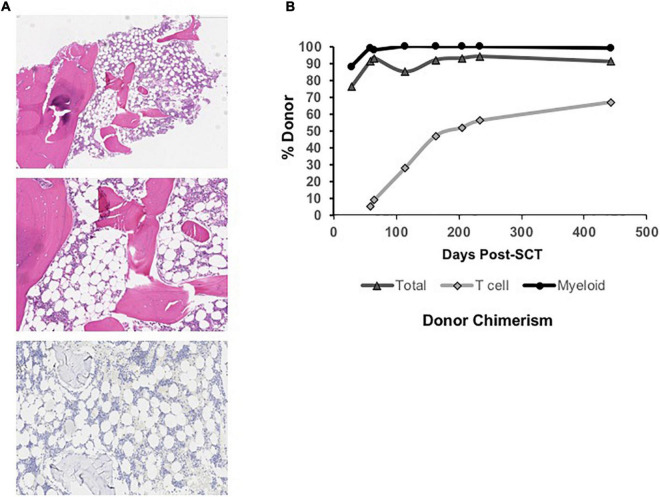
**(A)** Low power view (Top) of hypocellular bone marrow biopsy (∼30% cellular) taken at 26 months of age (4x, H, and E stain) in the patient with MPL-mutant CAMT. Higher power view (Middle) of hypocellular marrow with myeloid and erythroid hematopoiesis and absence of megakaryocytes (10x, H, and E stain). Immunohistochemical stain for CD42b (platelet receptor for von Willebrand factor) is negative (Bottom), confirming absent megakaryocytes (10x). **(B)** Total, T cell, and myeloid donor chimerism through 14 months post-bone marrow transplant showing evolution to complete myeloid donor chimerism and rising donor T cell chimerism over time.

At 26 months of age, ANC and Hgb remained normal, but red blood cell macrocytosis began to worsen (MCV increased from 75 to 90 fL). In addition, his platelet counts now averaged 10 K/μL with increased, although manageable, mucocutaneous bleeding. A repeat BM biopsy (Figure 1A) at this time showed evolving hypocellularity (30–40%) with absent megakaryocytes. We recommended proceeding to MSD-BMT at this time. While we considered using a busulfan-based myeloablative conditioning approach, the patient’s family wished to limit toxicity risks including infertility. We proceeded with a non-myeloablative conditioning regimen consisting of fludarabine 30 mg/m^2^/day from days –7 to –3; cyclophosphamide 60 mg/kg on days –5 and –4; thymoglobulin 3 mg/kg/day on days –3 to –1, as part of an institutional trial of fludarabine-based reduced toxicity conditioning for patients with iBMFS (NCT02928991). The patient received a T cell replete bone marrow graft from his sibling donor. He received EBV prophylaxis with rituximab (375 mg/m^2^) on day +1 and GVHD prophylaxis consisting of methotrexate 5 mg/m^2^/dose on days +1, +3, and +6, along with cyclosporine starting on day –1. As per institutional practice, cyclosporine was transitioned to tacrolimus starting on day +20, and tacrolimus was continued until a 2-month wean was initiated at 5 months post-transplant.

Neutrophil and platelet engraftment per Center for International Blood and Marrow Transplant Research (CIBMTR) criteria occurred on days +20 and +24, respectively (20). He required no further platelet or PRBC transfusions post-engraftment. He did not develop acute or chronic graft vs. host disease. He experienced no significant acute or chronic organ toxicity, including no pulmonary or hepatic complications and no significant viral reactivation or other infections. Immune reconstitution was excellent, and he was eligible for initiation of a revaccination schedule per institutional protocol by 8 months post-transplant. Initial peripheral blood donor chimerism was 76% overall on Day +28 with 88% myeloid donor chimerism (Figure 1B). Both subsequently improved, and he has maintained ≥ 99% myeloid donor chimerism and he has maintained 99% or greater myeloid donor chimerism since 4 months post-transplant. Donor T cell chimerism was initially low (5%) but improved to 67% at the last check, 14 months post-transplant.

## Discussion

CAMT is a rare autosomal recessive disorder most often caused by *MPL* mutations that presents with severe thrombocytopenia and absent megakaryocytes. Bleeding is often recognized at birth but may present several weeks later. Occasionally, CBC at birth may indicate normal platelet counts, and genetic testing is needed to confirm a diagnosis of CAMT (21). Coupled with the fact that CAMT has no physical abnormalities, diagnosis may be difficult without genetic testing or a family history (21).

The prognosis of patients with *MPL*-mutant CAMT is poor in the absence of alloSCT, with a median survival of 3 years (8). Most patients develop trilinear aplasia in the first decade of life. Clonal evolution to myelodysplasia and leukemia in *MPL*-mutant CAMT has been described (22), though recent cohort analyses suggest the actual incidence is low. The greatest risk for patients with *MPL*-mutant CAMT is progression to aplastic anemia that also requires alloSCT. The relatively low risk of hematopoietic clonal evolution compared to other IBMF syndromes, as well as the competitive advantage for donor HSCs over host HSCs, are important considerations supporting the use of aplastic anemia style conditioning regimens for alloSCT in *MPL*-mutant CAMT (2).

While TBI- and melphalan-based regimens have also been used, busulfan and cyclophosphamide approaches have been the most frequently utilized for patients with *MPL*-mutant CAMT, with reported survival rates of nearly 80% in patients receiving BMT from matched donors (14, 23). However, given that this patient population by definition consists of young pediatric patients, the long-term toxicities of myeloablative regimens, particularly concerning growth, pubertal development, and fertility, are concerning. Due to the young age at which patients with CAMT undergo alloSCT, sperm banking is not possible. There is little data on testicular tissue preservation and due to thrombocytopenia and evolving aplasia, there is an increased risk associated with this procedure in patients with CAMT. In contrast, non-myeloablative conditioning, such as cyclophosphamide/ATG-based regimens that are used for acquired aplastic anemia, is known to preserve fertility in a majority of patients, particularly those who are prepubertal at the time of transplant (24).

In conclusion, this report demonstrates that MSD-BMT using non-myeloablative conditioning for *MPL*-mutant CAMT can result in durable engraftment with complete myeloid donor chimerism. This approach should continue to be evaluated given the relative lack of experience with reduced intensity and non-myeloablative conditioning regimens in this patient population (14). An additional remaining question is whether this regimen would prove equally effective in alternative donor transplant approaches. Whether this non-myeloablative conditioning approach alters the optimal timing of transplant for patients with *MPL*-mutant CAMT and whether it can be successful before onset of BM hypoplasia require further consideration. The rarity of *MPL*-mutant CAMT likely precludes a prospective multi-center clinical trial and improved management will therefore benefit from continued reports on the refinement of these platforms for affected patients.

## Data availability statement

The original contributions presented in this study are included in the article/supplementary material, further inquiries can be directed to the corresponding author/s.

## Author contributions

JO and TO were clinically responsible for the patient, devised the plan of care and monitored the patient before, through and post-transplant, analyzed chimerism and other data, wrote and/or edited the manuscript. MP assisted with hematopathology and analyzed those data. KV took clinical care of patient on a day-to-day basis. YBS helped with data analysis and manuscript drafting. All authors contributed to the article and approved the submitted version.

## Abbreviations

AA: Aplastic Anemia
CAMT: Congenital Amegakaryocytic Thrombocytopenia
iBMFS: Inherited Bone Marrow Failure Syndromes
BM: Bone Marrow
Hgb: Hemoglobin
TPOR: Thrombopoietin Receptor
AlloSCT: Allogeneic Stem Cell Transplant
TBI: Total Body Irradiation
HSC: Hematopoietic Stem Cell
MSD BMT: Matched Sibling Donor Bone Marrow Transplant
ANC: Absolute Neutrophil Count
GVHD: Graft vs. Host Disease.
